# Tannic Acid Inhibits Hepatitis C Virus Entry into Huh7.5 Cells

**DOI:** 10.1371/journal.pone.0131358

**Published:** 2015-07-17

**Authors:** Shuanghu Liu, Ren Chen, Curt H. Hagedorn

**Affiliations:** 1 Department of Medicinal Chemistry, College of Pharmacy, University of Utah, Salt Lake City, UT, United States of America; 2 Department of Medicine, University of Utah, Salt Lake City, UT, United States of America; 3 Department of Medicine, University of Arkansas for Medical Sciences Little Rock, AR, United States of America; 4 Program in Genetics, University of Arkansas for Medical Sciences Little Rock, AR, United States of America; 5 The Central Arkansas Veterans Healthcare System, Little Rock, AR, United States of America; University of Washington, UNITED STATES

## Abstract

Chronic infection with the hepatitis C virus (HCV) is a cause of cirrhosis and hepatocellular carcinoma worldwide. Although antiviral therapy has dramatically improved recently, a number of patients remain untreated and some do not clear infection with treatment. Viral entry is an essential step in initiating and maintaining chronic HCV infections. One dramatic example of this is the nearly 100% infection of newly transplanted livers in patients with chronic hepatitis C. HCV entry inhibitors could play a critical role in preventing HCV infection of newly transplanted livers. Tannic acid, a polymer of gallic acid and glucose molecules, is a plant-derived polyphenol that defends some plants from insects and microbial infections. It has been shown to have a variety of biological effects, including antiviral activity, and is used as a flavoring agent in foods and beverages. In this study, we demonstrate that tannic acid is a potent inhibitor of HCV entry into Huh7.5 cells at low concentrations (IC_50_ 5.8 μM). It also blocks cell-to-cell spread in infectious HCV cell cultures, but does not inhibit HCV replication following infection. Moreover, experimental results indicate that tannic acid inhibits an early step of viral entry, such as the docking of HCV at the cell surface. Gallic acid, tannic acid’s structural component, did not show any anti-HCV activity including inhibition of HCV entry or replication at concentrations up to 25 μM. It is possible the tannin structure is related on the effect on HCV inhibition. Tannic acid, which is widely distributed in plants and foods, has HCV antiviral activity in cell culture at low micromolar concentrations, may provide a relative inexpensive adjuvant to direct-acting HCV antivirals and warrants future investigation.

## Introduction

Chronic hepatitis C virus (HCV) infection is a major cause of chronic liver disease and hepatocellular carcinoma (HCC) [1–3]. An estimated 3% of the world’s population is chronically infected with HCV (1). No vaccine is currently available; although treatments have undergone major improvements there remain needs for further advancements [4, 5]. Although HCV protease inhibitors and other direct-acting antiviral (DAA) agents have markedly improve the overall sustained virological response (SVR) following therapy, a significant proportion of patients with chronic hepatitis C remain unable to be treated with these regimens [6, 7]. The majority of new direct-acting antivirals target the replication step of HCV. Because of the high genetic heterogeneity of HCV and its rapid replication, monotherapy with DAA agents poses a high risk for selection of resistant variants and combinations of drugs targeting different steps of the viral life cycle, including virus entry, would likely improve viral response rates across a wider range of HCV genotypes and clinical settings [8].

HCV is a member of the Flaviviridae, has a 9.6 kb positive-stranded RNA genome, encodes for a single polyprotein cleaved by cellular and viral proteases into 10 different proteins: core, E1, E2, p7, and the nonstructural proteins, NS2, NS3, NS4A, NS4B, NS5A, and NS5B [9, 10]. The E1 and E2 (envelope) glycoproteins play a central role in virus entry into the hepatocytes which is a complex multistep process [11, 12]. At least four entry factors, including scavenger receptor class B type 1, tetraspanin cluster of differentiation (CD) 81, claudin-1, and occluding are sequentially involved after virus binding and HCV entry is via clathrin-mediated endocytosis [13, 14]. Attractive targets for cell entry antivirals include blocking virus-target cell interactions during attachment, post-binding events or viral fusion, any of which could provide complementary mechanisms of action to DAAs [15, 16].

HCV pseudo-particles, which consist of retroviral or lentiviral cores surrounded by an envelope containing HCV E1 and E2, have provided a valuable system to study viral and cellular determinants of the entry pathway [17, 18]. The establishment of an infectious HCV cell culture system (HCVcc) with a genotype 2a isolate (JFH1 strain) of HCV and Huh7 cells was critical in better understanding HCV entry [19, 20]. These systems allowed a number of HCV entry inhibitors to be identified [21–24], such as anti-CD81 antibodies and entry inhibitor 1 (EI-1) which blocks viral fusion [22,24].

**Fig 1:**
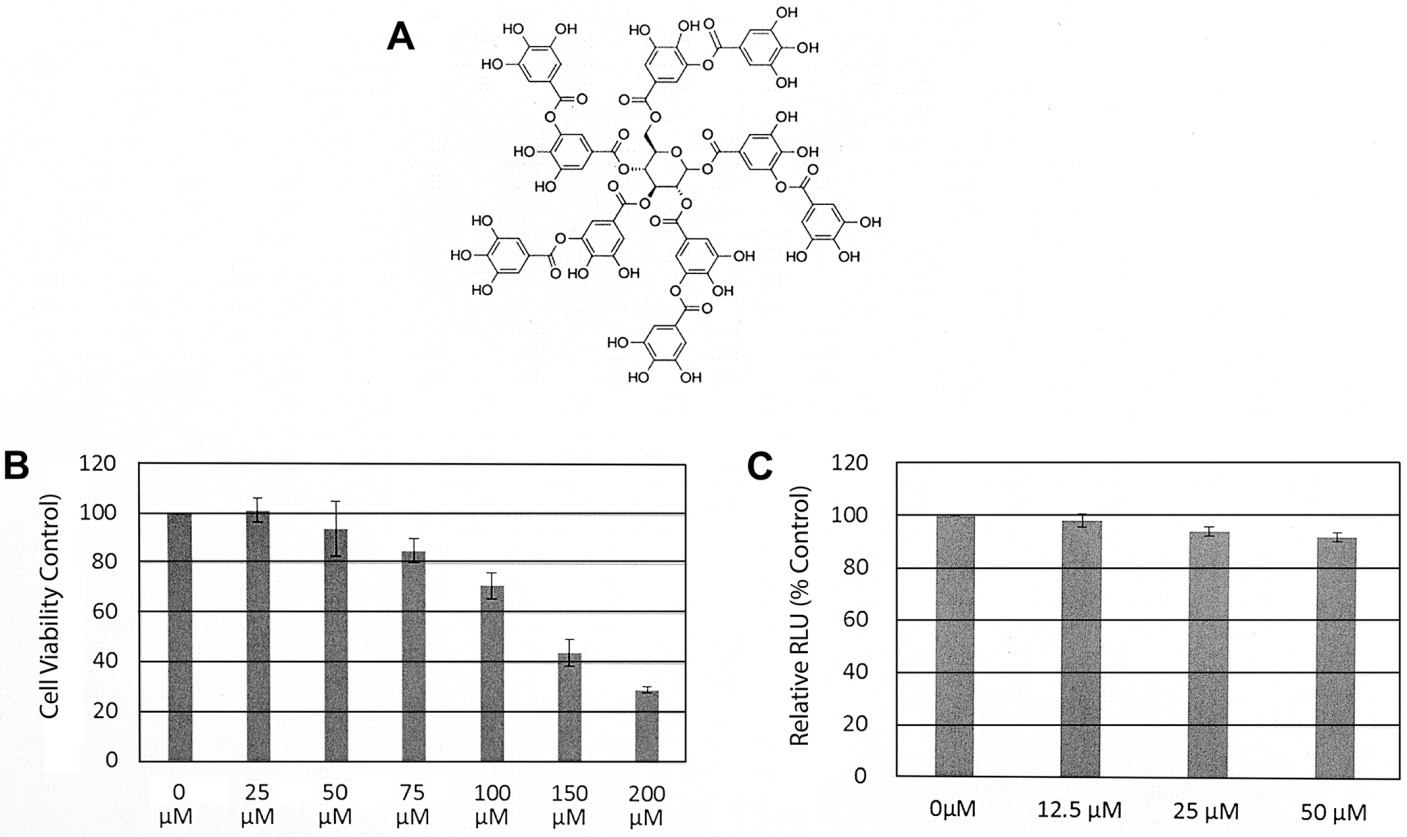
The structure of tannic acid and toxicity assays. (A) Molecular structure of tannic acid. (B) Cell toxicity of tannic acid as measured by cell viability. The viability of Huh7.5 cells was determined using a CellTiter 96 Aqueous One solution cell proliferation assay as described in Materials and Methods. Each experiment was performed in triplicate and repeated at least three times. The CC_50_ based on these studies was 146.1 ± 6.2 μM. (C) The direct effect of tannic acid on Renilla luciferase (Rluc) assays: HCV JFH1-AM120-Rluc infected cell lysates containing Rluc protein was mixed with increasing concentrations of tannic acid for ten minutes at room temperature and luciferase activity was measured as described in Materials and Methods. Assays were done in triplicate and experiments done three times. Results are presented as the mean ± standard deviation (SD) (n = 9) of relative light units (RLU). No significant difference (Student’s t-test p>0.05) in Rluc assay results were observed with tannic acid concentrations ≤ 50 μM.

Tannic acid is a plant-derived hydrolysable tannin polyphenol that is a gallic acid polymer glucoside (C_76_H_52_O_46_, 1,701.20 Da) (Fig 1A) [25]. It is widely distributed in the plant kingdom, including food grains, fruits, herbs, vegetable and beverages such as tea, red wine, and coffee [26–28]. Tannic acid has been claimed to have a variety of beneficial effects on health that are believed to be primarily related to its antioxidant properties [29, 30]. Tannic acid inhibits the proliferation of different cancer cell lines [31, 32] and induces cancer cell apoptosis [33–35]. It enhanced the survival rate of mice bearing syngeneic tumors when given in drinking water [36]. Other studies have shown that tannic acid prevents azidothymidine (AZT) induced hepatotoxicity in mice [37]. Antiviral activities of tannic acid have been reported and are generally thought to be due to interference with viral adsorption to the host cell membrane and not antioxidant properties (e.g., HIV, Bovine adeno-associated virus and Noroviruses) [31, 38–40] In addition a recent report showed tannic acid can inhibit the attachment of Influenza A and Human papillomavirus at relatively low concentrations (41). However, it is unknown whether it has any effect against HCV.

The green-tea polyphenol, epigallocatechin-3-gallate (EGCG), which is one type of tannin, was reported to inhibit HCV entry [42, 43]. Tannic acid, a common tannin, is structurally similar to EGCG [25, 26, 44, 45]. We hypothesized that tannic acid may have anti-HCV activities and tested this hypothesis in an infectious HCV cell culture system. In this report we provide evidence that tannic acid inhibits HCV entry and cell-to-cell transmission but does not interfere with intracellular HCV replication. Tannic acid is widely distributed in food and beverages, is present in blood and tissues, and may be a natural barrier to HCV infection.

## Materials and Methods

### 2.1 Cell culture

Human hepatoma cells, Huh7.5, were generously provided by Dr. Charles M. Rice [46] and maintained in Dulbecco's modified Eagle's medium (DMEM) (Invitrogen) supplemented with 100 U/ml of penicillin, 100 μg/ml of streptomycin, nonessential amino acids, and 10% fetal bovine serum (FBS) (Invitrogen) at 37°C in 5% CO_2_. All experiments described in this study were performed using these cells.

### 2.2 Antibodies

The monoclonal antibody to the NS5A protein (9E10) was a gift from Dr. Charles Rice (Rockefeller University). The monoclonal antibody against the HCV NS3 protein (Abcam, MA, USA), goat anti-mouse conjugated with horseradish peroxidase (HRP) (Sigma) and goat anti-mouse IgG conjugated with Alexa Fluor 594 (Invitrogen) were obtained commercially.

### 2.3 Test compounds

Tannic acid was obtained from MP Biomedicals LLC, Solon, OH, USA and Post Apple Scientific Inc., North East, PA, USA. Tannic acid was dissolved in deionized water and sterilized using a 0.22 μM filter unit (Millipore) and 5 mM stock solutions were prepared. Heparin and gallic acid were purchased from Sigma-Aldrich and dissolved in water and sterilized in the same way to produce stock concentrations of 500 mg and 5 mM, respectively

### 2.4 Preparation of stock HCV JFH1-AM120 Renilla luciferase (JFH1-AM120-Rluc) reporter virus

The JFH1-AM120-Rluc reporter virus has been described previously [19, 47]. To generate the full-length genomic RNA, JFH1-AM120-Rluc was linearized at the 3' end of the HCV cDNA with *Xba*I. The linearized plasmid DNA was purified and used as a template for T7 *in vitro* transcription (MEGAscript, Ambion). *In vitro* transcribed RNA was transfected into cells by electroporation as described previously [48, 49]. Media containing virus was collected, clarified by low speed centrifugation (1,500 x g for 10 minutes), and stored at -80°C. HCV titers were determined by infection of Huh7.5 cells with serial dilutions of virus, followed by indirect immunofluorescence for HCV NS5A protein as described below and expressed as focus forming units (ffu)/ml. Several passages of infected cells were required to generate high titer stocks of virus.

### 2.5 Immunofluorescence Assays (IFA)

Cells infected with HCV were washed with PBS, fixed with 4% paraformaldehyde, and permeabilized with 0.2% Triton X-100. Fixed cells were blocked with 1% bovine serum albumin and 1% normal goat serum in PBS. HCV NS5A protein in cells was detected by incubation with an NS5A-specific monoclonal antibody and visualized with the secondary goat anti-mouse IgG conjugated to Alexa Fluor 594 dye (Life Technologies, 1:1,000 dilutions). Cover slips were mounted onto slides with DAPI (Vector Labs) and the HCV proteins were visualized by fluorescence microscopy (Nikon E400).

### 2.6 Titration of Infectious HCV

The titer of infectious HCV preparations was determined by IFA assays of inoculated cells, where the number of NS5A positive cell foci were determined as previously described [20]. Cell supernatants were serially diluted 10-fold in DMEM. The supernatants were used to infect 1x10^4^ naïve Huh7.5 cells in 96-well plates. Cells were incubated with virus for 2h at 37°C, washed and incubated with complete DMEM. The level of HCV infection was determined three days post infection by immunofluorescence staining for NS5A. Viral titers are expressed as focus-forming units per milliliter of supernatant (ffu/ml).

### 2.7 Western Blot Analysis

The HCV infected or transfected Huh7.5 cells were lysed in a radioimmunoprecipitation assay (RIPA) buffer (50 mM Tris-HCl, pH 7.5, 150 mM NaCl, 1% Nonidet P40, 0.5% sodium deoxycholate) containing a cocktail of protease inhibitors (Roche). The total protein for each sample was measured using a standard protein assay (Bio-Rad). Twenty micrograms of total protein of each sample was analyzed by 8% SDS-PAGE and transferred to nitrocellulose membranes. The membranes were blocked with 5% skim milk. HCV proteins were detected with a monoclonal antibody specific for NS3, horseradish peroxidase-conjugated goat anti-mouse immunoglobulin G (Bio-Rad) and a chemiluminescence substrate (Pierce). Actin was used as a control and was detected with an anti-β-actin monoclonal antibody (Sigma).

### 2.8 The Effect of Tannic Acid on HCV 1b Replicons

The HCV 1b replicon Con1/SGNeo (I) plasmid was a gift from Dr. Charles Rice (Rockefeller University) [50]. The plasmid was linearized with *Sca*I and *in vitro* transcription was performed as described previously [46]. HCV 1b replicon RNA was electroporated into Huh7.5 cells and seeded into six-well plates and cultured for 48 hours. Cells were trypsinized, subcultured into twelve-well plates and the tannic acid at concentrations of 0 μM, 3.1 μM, 6.3 μM, 12.5 μM and 25 μM was added the following day. The cells were then culture with tannic acid for 48 hours. Mock electroporation (no HCV1b replicon RNA) of cells provided controls. Cells were lysed in RIPA buffer and Western blots were done with anti-NS3 monoclonal antibodies.

### 2.9 Quantification of HCV RNA by qPCR

Huh7.5 cells in twelve-well plates were infected with HCV JFH1-AM120-Rluc for 2 hours in the presence of the described concentrations of tannic acid; cells were washed with PBS and were incubated for 48 hours under standard conditions. Total RNA was extracted with Trizol (Invitrogen) and HCV RNA was measured by qPCR analysis as described previously [51]. The relative quantity of HCV RNA in control and HCV samples was calculated using the comparative Ct (cycling threshold) method. A reference gene (β-actin) was used as a control.

### 2.10 Renilla Luciferase Reporter Assay for the inhibition of HCV infection

Huh 7.5 cells were grown overnight in 96-well plates under standard conditions. Cells were infected for 2 hours with HCV JFH-AM120-Rluc at a multiplicity of infection (MOI) of 0.1 with or without compounds. At 48 hours post-inoculation the cells were treated with lysis buffer at room temperature for 15 minutes. Renilla luciferase (Rluc) activity was measured in cell lysates using a Renilla luciferase Assay System kit (Promega) and microplate reader (Bio-Tek). Normalized luciferase activity was plotted as a function of inhibitor concentration and IC_50_ values were calculated from a linear fit of the inhibitor data.

### 2.11 Toxicity assays of tannic acid

Cell viability was determined using a CellTiter 96 Aqueous One solution cell proliferation assay kit (Promega) following the manufacturer’s instructions. Briefly, aliquots of 1 × 10^4^ Huh7.5 cells/well were cultured in 96-well plates with fresh medium or medium with increasing concentrations of Tannic acid for 48 and 72 hours. CellTiter 96 Aqueous One solution (40 μl, Promega) was added to each well and incubated for 3 hours. The absorbance at 490 nm was measured with a 96-well plate reader. Each experiment was performed in triplicate and repeated at least three times. The concentration of 50% cellular cytotoxicity (CC_50_) of tannic acid was determined as the drug concentration that yielded 50% cell death using MS-Excel software. Possible direct effects of tannic acid on Renilla luciferase was examined as follows. JFH1-AM120-Rluc infected cell lysates containing Renilla luciferase were mixed with increasing concentrations of tannic acid and incubated for 10 minutes at room temperature and luciferase activity was measured as described above. Assays were done in triplicate and experiments done three times.

### 2.12 Quantitative Binding Assay

Huh7.5 cells were inoculated with HCV JFH1AM120-Rluc at a MOI of 10 for 1 hour at 4°C in presence of 50 μM of tannic acid, or 500 μg/ml of heparin, no inhibitor as a control. Cells were washed twice with ice-cold PBS, and total RNA was extracted using the Trizol (Invitrogen) according to the manufacturer’s instructions. HCV RNA was quantified by quantitative real-time reverse transcription polymerase chain reaction (qRT-PCR) assay as described previously [51].

### 2.13 Entry Assay

JFH1-AM120-Rluc virus binding to target Huh7.5 cells was performed for 1h at 4°C in the absence or in the presence of compounds in 24-well plates. Subsequently, cells were washed with PBS and shifted to 37°C to allow entry to proceed. Depending on the protocol, inhibitors were added directly or 1, 2 or 3 h thereafter (II, III, and IV, respectively) and incubated in complete culture medium at 37°C for 48 hours. Infections were scored by measuring luciferase activity.

### 2.14 HCV Cell-to-Cell Transmission Assay

An assay incorporating a semisolid medium was used to measure cell-to-cell spread of HCV infection [52]. HCV was added to Huh7.5 cells at a MOI of 0.005 ffu/cell and incubated at 37°C. After three hours of infection, the inoculum was removed and replaced with culture medium containing 1% low melting temperature agarose (Invitrogen) and 25 μM tannic acid or an equivalent volume of water as a control. To prevent the agarose from drying, 200 μl of complete DMEM was used to cover the agarose layer. Cells were incubated at 37°C for 3 days at which time the agarose was removed and the infected cells were analyzed by indirect immunofluorescence for the HCV NS5A as described above. The mean number of infected cells/ffu was determined from 20 foci.

### 2.15 Statistical analysis

The mean and standard deviation of the mean (SD) for datasets were determined and the results for different experimental conditions (e.g., inhibitor or no inhibitor) were compared using the Students *t*-test. A *P* value < 0.05 was considered to be significant.

## Results

### 3.1 Toxicity Assays of Tannic Acid

Tannic acid (C_76_H_52_O_46_) is a polyphenol that is highly soluble in water (Fig 1A). Before testing the hypothesis that tannic acid had HCV antiviral effects, we first determined the cytotoxicity of tannic acid on Huh7.5 cells. Cytotoxicity was measured using a CellTiter 96 Aqueous One solution cell proliferation assay kit (Promega) according to the manufacturer’s instructions. Tannic acid at concentrations of less 50 μM showed no toxic effects on cells after 48 hours of incubation (Fig 1B). The CC_50_ of tannic acid for cultured Huh7.5 cells was 146.1 ± 6.2 μM at 48 and 126.9±15.3 μM at 72 hours. Although luciferase reporter enzymes can speed the evaluation of antiviral compounds, they can also be directly inhibited by some compounds leading to false positives in screening tests [53]. To determine whether tannic acid directly inhibited Rluc activity, lysates of JFH1-AM120Rluc infected cells expressing Rluc protein was mixed with increasing concentrations of tannic acid for 10 minutes at room temperature and luciferase activity was measured. The result showed that tannic acid did not directly inhibit Rluc activity at concentrations less than 50 μM (Fig 1C).

### 3.2 Effect of Tannic Acid on HCV Infection

**Fig 2:**
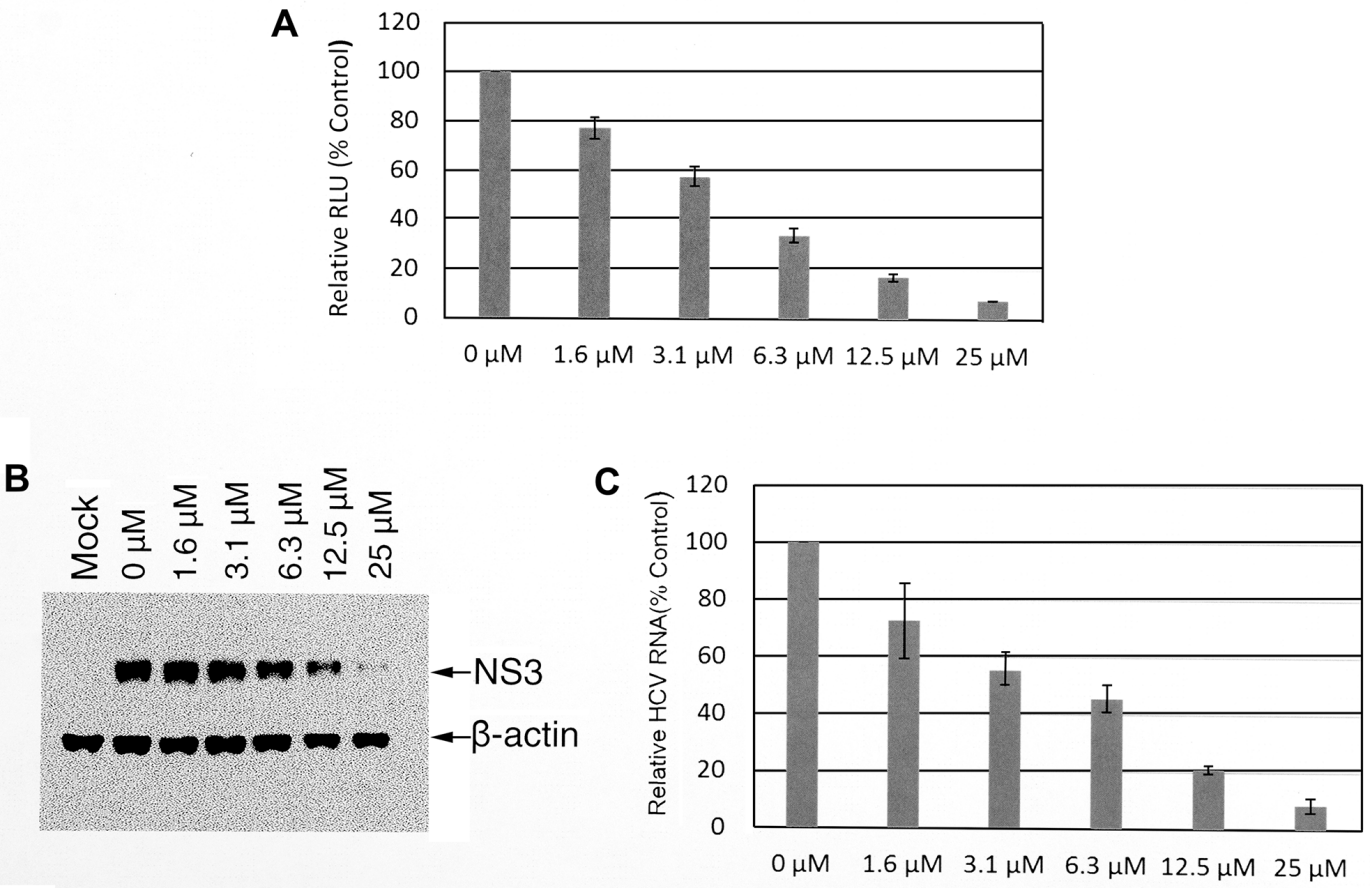
Tannic acid inhibits HCV infection. (A) The effect of tannic acid on an infectious HCV cell culture system. Huh7.5 cells in 96 well plates were infected with HCV JFH1-AM120-Rluc for two hours in the presence of the specified concentrations of tannic acid, cells were washed with PBS and incubated for 48 hours. Cells were lysed and luciferase activity was measured as described in Materials and Methods. The results for three experiments are shown as mean RLUs ± SD of triplicate cell culture assays in 96-well plates. The IC_50_ was 5.8 ± 0.38 μM. (B) The effect of tannic acid on the production of HCV NS3 protein. Huh7.5 cells were infected with JFH1-AM120-Rluc for two hours in the presence of the specified concentrations of tannic acid, cells were washed with PBS and incubated for 48 hours. Cells were lysed and Western blotting was done with anti-HCV NS3 antibodies as described in Materials and Methods. The image represents three different experiments. (C) The effect of tannic acid on the production of HCV RNA. Huh7.5 cells were infected with JFH1-AM120-Rluc for two hours in the presence of the specified concentrations of tannic acid, cells were washed with PBS and incubated for 48 hours. The total RNA was extracted and qPCR assays for HCV RNA were done as described in Materials and Methods. Error bars represent the mean ± SD of results from three experiments.

The structure of tannic acid is similar epigallocatechin-3-gallate epigallocatechin-3-gallate (EGCG) and previous studies have demonstrated EGCG has anti-HCV activity by blocking HCV entry [30, 54, 42, 43]. We hypothesized that tannic acid may have a similar effect on HCV. To confirm this hypothesis, we used an infectious chimeric HCV cell culture system expressing Renilla luciferase (JFH1-AM120-Rluc) to infect cells (MOI = 0.1) in the presence of increasing concentrations of tannic acid [47]. After two hours of infection, the inoculum was removed; cells were washed with PBS and incubated for 48 hours. Cells were lysed and luciferase activity was measured as described in Materials and Methods. Tannic acid produced a dose-dependent decrease in HCV infection as measured by Rluc activity (Fig 2A). The inhibitory effect of tannic acid was also tested with HCV at an MOI of 0.01, 0.2 and 0.5. The results showed a similar dose response with this range of MOIs (Data not showed). The IC_50_ for tannic acid was 5.8 ± 0.38 μM and the therapeutic index was approximately 25. To verify that changes observed after tannic acid treatment indeed reflected changes in HCV infection, the HCV NS3 protein and HCV RNA were measured after HCV infection and tannic acid treatment by Western blotting and qPCR, respectively. Both of the NS3 protein (Fig 2B) and HCV RNA levels (Fig 2C) decreased in a dose dependent manner that corresponded to changes in Rluc activity, supporting the antiviral activity of tannic acid. Similar results were found when tannic acid from two other suppliers was tested (see Materials and Methods) (data not shown).

### 3.3 Tannic Acid Inhibits an Early Step of HCV Infection

**Fig 3:**
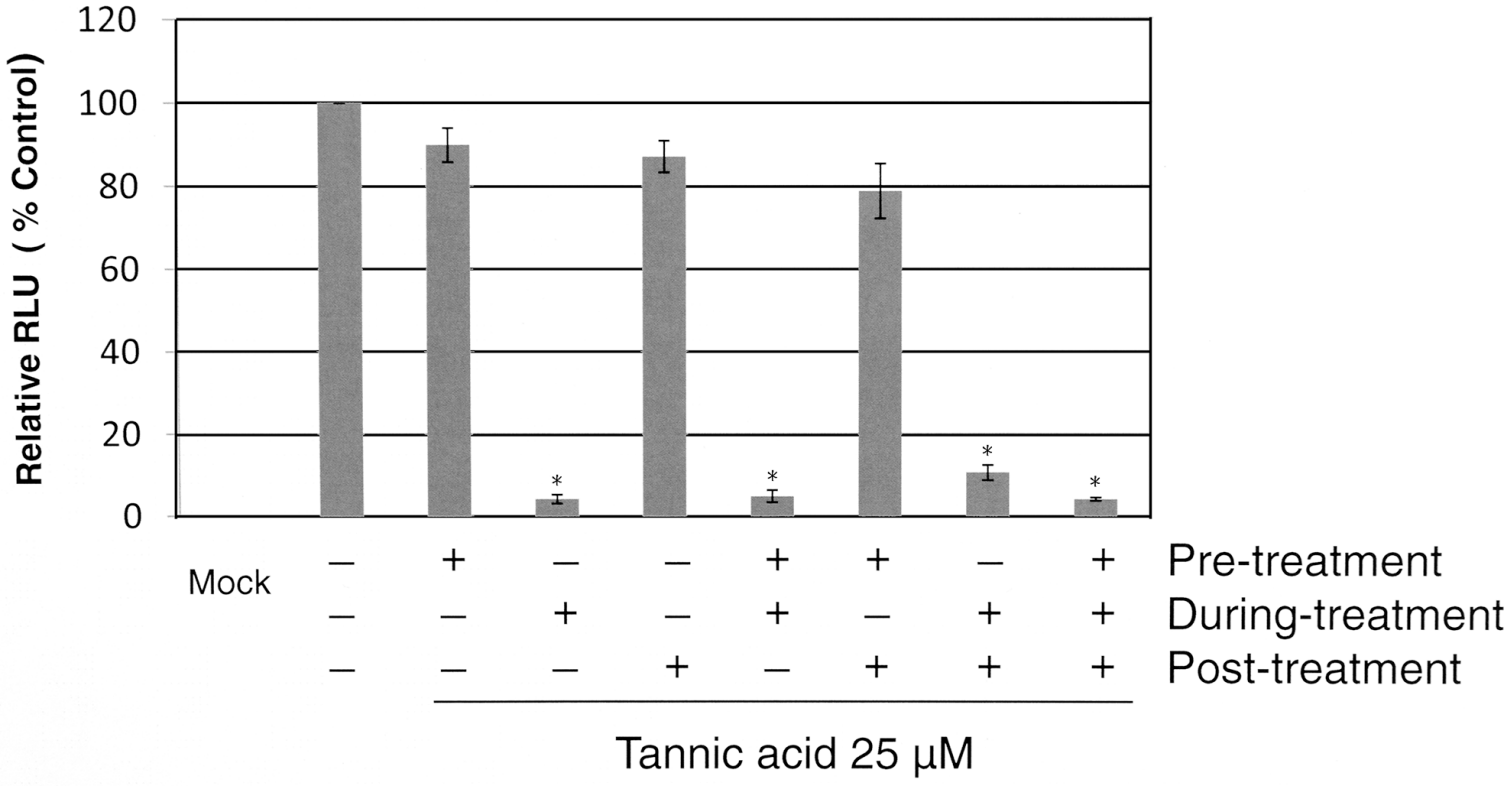
Tannic acid inhibits an Early Step of HCV Infection. Tannic acid 25μM was added to the culture medium of Huh7.5 cells for two hours before infection (pre-treatment), for two hours during infection (during-infection), and for two hours after infection with JFH1-AM120Rluc virus (post infection). Forty-eight hours post infection, cells were lysed and luciferase activity was measured. Infectivity is expressed as a percentage relative to luciferase activity measured in controls without tannic acid. Mock infection was the negative control. Mean values ± SD of three different experiments done in triplicate are presented. Results with a Student’s *t*-test P value < 0.01 are indicated by an asterisk.

To identify which step of HCV infection tannic acid inhibited, tannic acid 25 μM was added to Huh7.5 cells before, during, and after inoculation with HCV JFH1-AM120-Rluc. Tannic acid was added to the culture medium of Huh7.5 cells for 2 hours before JFH1-AM120-Rluc virus infection (pretreatment), for two hours during infection with the JFH1-AM120-Rluc virus (during infection), and for two hours after infection with JFH1-AM120-Rluc (post infection). Forty-eight hours post infection, cells were lysed and luciferase activity was measured. Infectivity is expressed as a percentage relative to the luciferase activity measured in controls not treated with tannic acid. Mock Infection with conditional medium served as a negative control. Interestingly, HCV infection was inhibited only when tannic acid was present during virus infection and tannic acid had no effect if added before or after HCV infection (Fig 3). These results suggested that tannic acid inhibited a relatively early step in the HCV life cycle, such as viral entry.

### 3.4 Effect of Tannic Acid on HCV Replication

**Fig 4:**
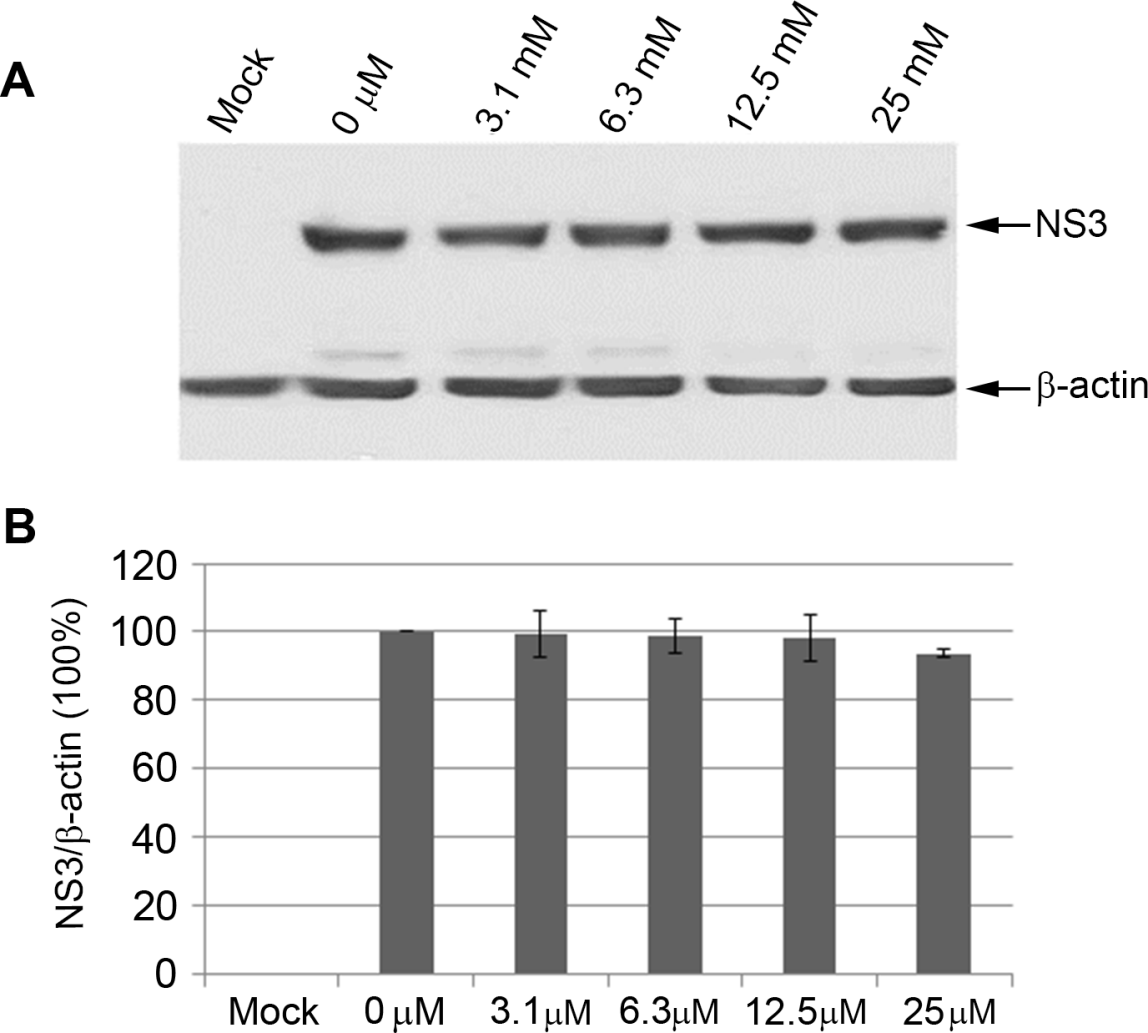
Tannic acid does not inhibit HCV replication. (A) The effect of tannic acid on a HCV replicon, that displays intracellular replication events, was determined. HCV1b replicon RNA was electroporated into Huh7.5 cells followed by seeding of cells into six-well plates. After 48 hours of culture cells were trypsinized and seeded into twelve-well plates and tannic acid was added at concentrations of 0 μM, 3.1 μM, 6.3 μM, 12.5 μM and 25 μM and cells were cultured for 48 hours as described in Materials and Methods. Mock electroporation (no HCV1b replicon RNA) of cells provided controls. Cells were lysed and Western blots were done with anti-NS3 monoclonal antibodies as described in Materials and Methods. Experiments were performed three times and representative examples are shown. (B) The levels of NS3 were normalized to β-actin and the ratios of NS3/β-actin relative to 0 μM tannic acid (no inhibitor, 100% level of NS3) were plotted against inhibitor concentrations. The data are presented as mean ± SD (n = 3) of the NS3 levels normalized to β-actin for three independent experiments.

After determining the antiviral activity of tannic acid in an infectious HCV cell culture system, we sought to analyze the antiviral mechanism of tannic acid using a sub-genomic HCV 1b replicon cell culture system. This system only displays HCV intracellular replication and does not exhibit viral entry, assembly and release steps. HCV 1b replicon RNA was electroporated into Huh7.5 cells and seeded to wells in twelve-well plates. Six hours post-electroporation, tannic acid at concentrations of 0 μM, 3.1 μM, 6.3 μM, 12.5 μM and 25 μM was added and cells were cultured for 48 hours. Mock electroporation (no HCV 1b replicon RNA) of cells provided control. Cells were lysed and Western blots were done with anti-NS3 monoclonal antibodies as described in Materials and Methods. Immunoblotting for HCV NS3 showed no decrease at the concentrations of tannic acid tested (Fig 4), providing evidence that tannic acid does not inhibit HCV replication.

### 3.5 Tannic Acid Blocks an Early Step of HCV Entry

**Fig 5:**
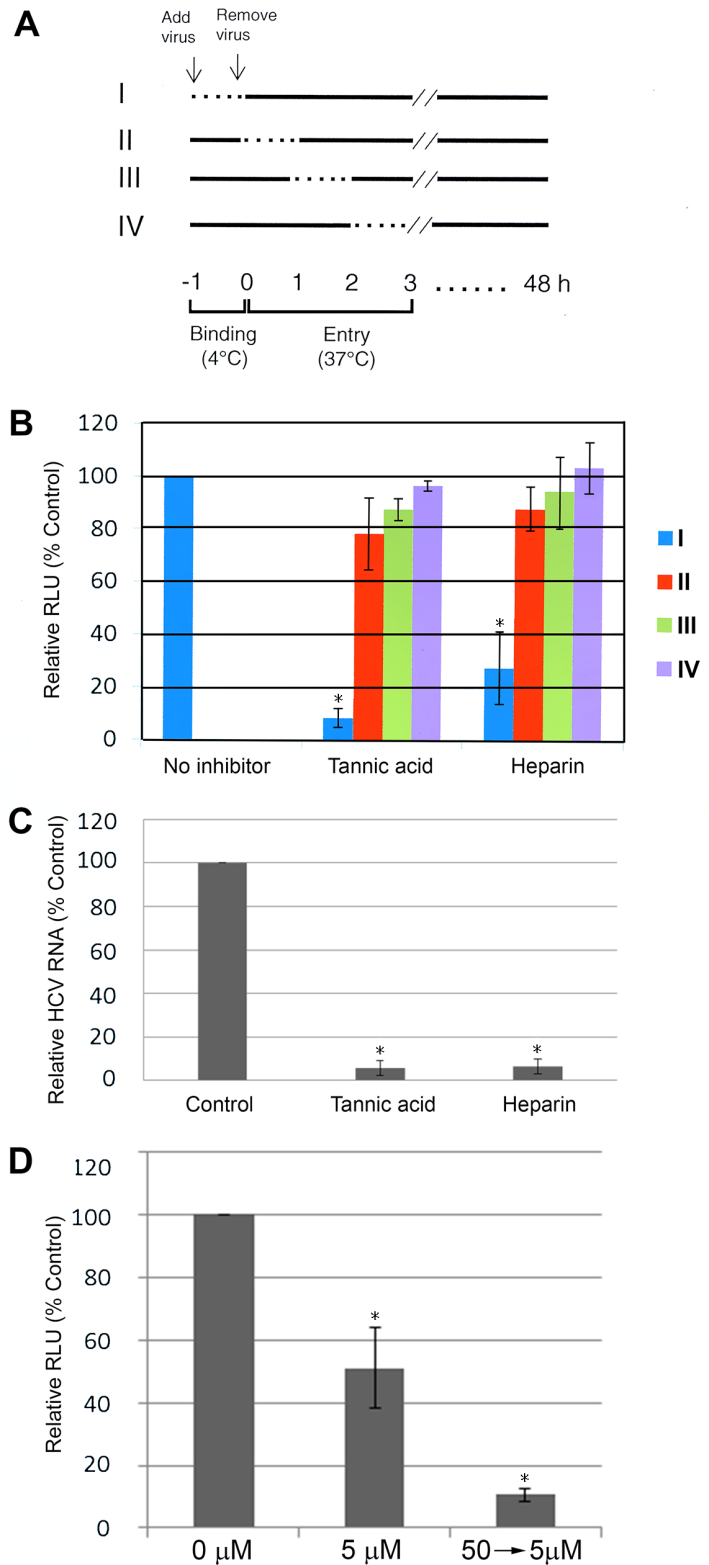
Tannic acid blocks an early step of HCV entry. (A) Schematic drawing of the experiment design. Inhibition of HCV JFH1-AM120-Rluc entry into Huh7.5 cells by tannic acid (25 μM) and heparin controls (200 μg/ml) was compared using four different experimental conditions (indicated by I through IV). Virus binding to cells was performed for 1 h at 4°C in the absence (II to IV) or presence (I) of compounds. Subsequently, cells were washed with PBS and shifted to 37°C to allow HCV entry to proceed. Inhibitors were either added directly, at 1,2 or 3 hours as indicated (II, III and IV, respectively). Dotted lines indicate the time that an inhibitor was present; black arrows indicate the addition and removal of the HCV inoculum. (B) Effect of different times of tannic acid or heparin treatment on HCV infection. The experimental design describe in Panel A was followed, luciferase assays were done at 48 hours post infection and measures of infection are expressed relative to HCV infection in the absence of inhibitors. The data are presented as mean ± SD of three experiments done in triplicate. Results with a Student’s *t*-test P value < 0.01 are indicated by an asterisk. (C) Effect of tannic acid or heparin treatment on HCV binding to cells as measured by HCV RNA. Huh7.5 cells were inoculated with HCV JFH1-AM120-Rluc at a MOI of 10 for one hour at 4°C in presence of 50 μM of tannic acid, 500 μg/ml of heparin or no inhibitor as a control. Cells were washed twice with ice-cold PBS and total RNA was extracted. HCV bound to cells was measured by qPCR analysis of HCV RNA. Relative binding is expressed as the percentage of the control for (100%). Results are shown as the mean ± SD of three different experiments done in triplicate. Results with a P value < 0.01 are indicated by an asterisk. (D) Effect of preincubating HCV with tannic acid on their infectivity. JFH1-AM120-Rluc pre-treated with 50 μM tannic acid for 15 minutes and then diluted to decrease the concentration of tannic acid to 5 μM prior to the inoculation of Huh-7.5 cells. Cells were inoculated for two hours with HCV JFH1-AM120-Rluc was preincubated with tannic acid (as described) or without tannic acid then incubated with (5 μM) or without tannic acid as controls. The virus titers were kept constant in the three different conditions. At 48 hours post infection, cells were lysed and luciferase activity was measured. Infectivity is expressed as the percentage of the control (no tannic acid) which was assigned a 100% value. Mean values ± SD of three independent experiments done in triplicate are presented. Results with a P value < 0.05 are indicated by an asterisk.

The mechanism of HCV entry is a complex multistep process involving binding of viral particles to the cell surface, interaction with entry factors followed by endocytosis and fusion of the viral envelope with an internal membrane [12]. Because our initial analysis provided evidence that tannic acid inhibited an early step of the HCV life cycle, we conducted studies aimed at identifying which step of viral entry was impaired. Previous studies demonstrated that at 4°C HCV attaches to cells but does not efficiently enter cells and at 37°C the virus enter cells via endocytosis and membrane fusion [21]. To determine which step of HCV entry was impaired, tannic acid was added at different times when HCV JFH1-AM120-Rluc was allowed to bind to Huh7.5 cells at 4°C (1 hour) and cells were subsequently incubated at 37°C (Fig 5A). Heparin, a known inhibitor of HCV binding, was used as a positive control [21]. A strong decrease in HCV infection was observed when tannic acid was present during the one hour 4°C binding step, whereas no inhibition was observed when tannic acid was added after virus binding. As expected, similar results were obtained when heparin was added during the 4°C binding step, but had no effect if added following this step (Fig 5B).

To determine whether tannic acid directly impaired the binding of HCV particles to the cell surface or a later step of virus entry, we analyzed virus binding in the presence of tannic acid. Huh7.5 cells were inoculated with HCV JFH1-AM120-Rluc for one hour at 4°C in the presence of tannic acid or heparin and inoculated cells without inhibitors provided controls. After one hour cells were washed with PBS, RNA was extracted from cells using Trizol and the amount of bound HCV was measured by qPCR assays of HCV RNA. As expected, heparin strongly reduced HCV attachment to the cell surface as measured by HCV RNA qPCR assays (Fig 5C). In the presence of tannic acid, a marked decrease in virus binding was also observed as compared to controls.

These results provide evidence that tannic acid most likely has an antiviral effect by inhibiting the binding of HCV to the cell surface.

To further investigate if tannic acid affects viral particles or cells, infectious HCV particles were preincubated with tannic acid before their use in inoculating Huh7.5 cells. If tannic acid acts directly on HCV particles, the preincubation treatment of HCV was predicted to have a marked inhibitory effect on infection. Preincubation of the virus with 50 μM of tannic acid, followed by dilution to 5 μM during infection, led to a stronger inhibition of infection than that observed with 5 μM of tannic acid during inoculation without any preincubation (Fig 5D). These data provide evidence that tannic acid inhibits HCV entry by a direct effect on HCV particles.

### 3.6 Effect of Tannic Acid on Cell-to-cell Spread of HCV

**Fig 6:**
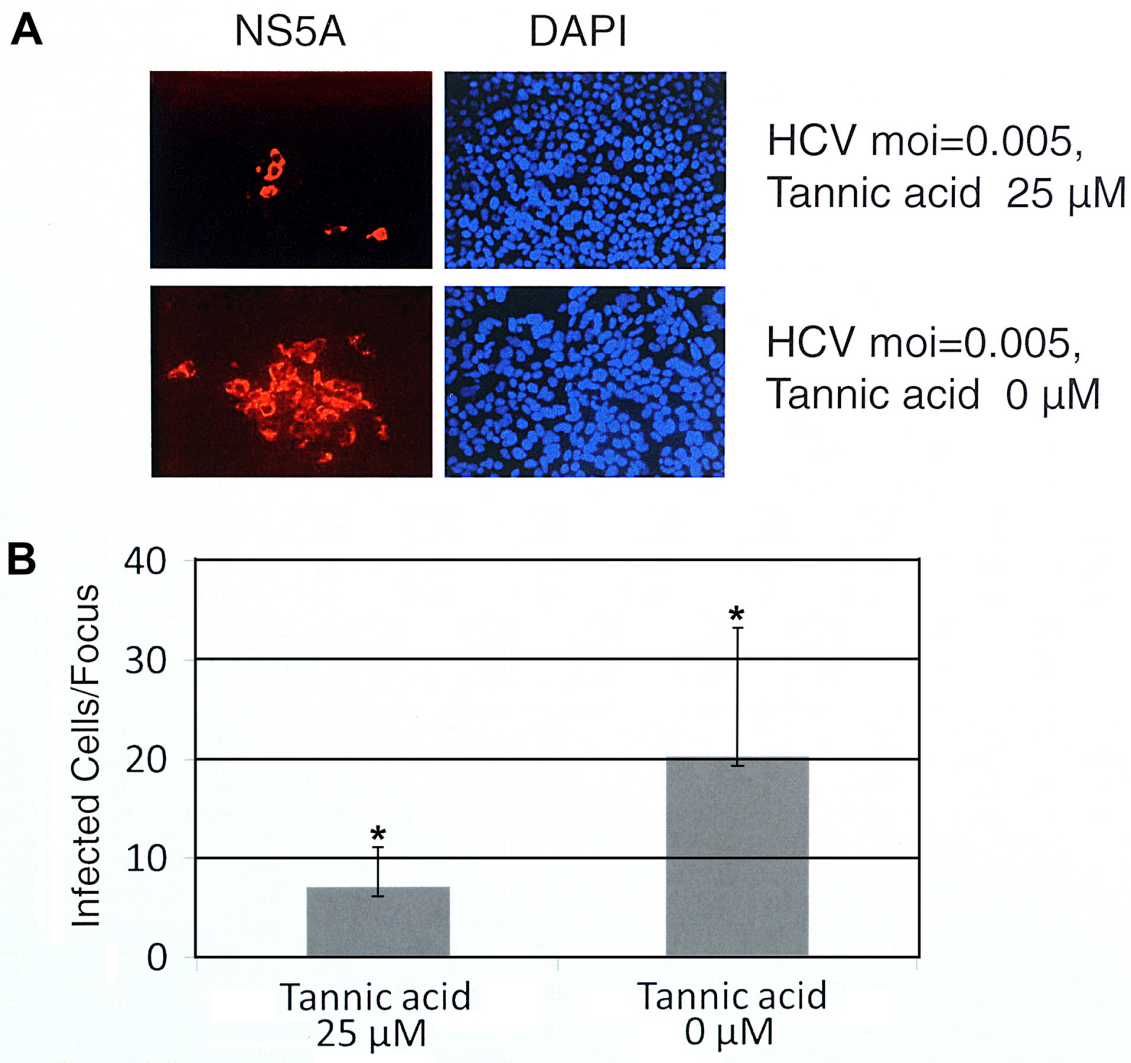
Effect of tannic acid on cell-to-cell spread of HCV. (A) The effect of tannic acid on cell-to-cell spread of HCV as measured by viral protein staining. Huh7.5 cells were infected with HCV using an MOI of 0.005 at 37°C. The inoculum was removed two hours post infection and replaced with a 1% agarose medium overlay containing 25 μM tannic acid. Controls had no tannic acid. Cells were incubated at 37°C for 72 hours as described in Materials and Methods. Infected cells were labeled by indirect immuno-fluorescence using an anti-HCV NS5A monoclonal antibody (red), nuclei were stained with DAPI (blue) and photographs were taken with a fluorescence microscope as described in Materials and Methods. (B) The mean and standard deviation of infected cells/focus was determined by visual counting of 30 foci present on randomly selected fields of cover slips for each condition. P-values were calculated using the Student’s ttest (asterisk indicates P = 0.000019). Experiments were performed three times and representative examples are shown in A and B.

Following infection of Huh7.5 cells with HCV the transmission of progeny virus to adjacent cells results in localized collections of infected cells called foci. To determine whether tannic acid could block the cell-to-cell spread of HCV, Huh7.5 cells were infected with an MOI 0.005 of HCV. At two hours post infection the inoculum was removed and replaced with culture medium containing 1% low melting temperature agarose with tannic acid 25 μM or without tannic acid (controls). Cells were incubated at 37°C for three days at which time the agarose was removed and the infected cells were detected using immunofluorescence assays for the HCV NS5A protein as described in Materials and Methods. Foci were visualized by immunofluorescence microscope (Fig 6A) and size of foci was measured by counting the number of cells per foci present in randomly selected fields for each condition (Fig 6B). Tannic acid produced a marked reduction in the number of cells per focus forming unit (average of seven cells per focus) as compared to the control conditions (average of 20 cells per focus) (p = 0.00058). These results provide evidence that tannic acid prevents *in vitro* HCV cell-to-cell transmission.

### 3.7 Effect of Gallic Acid on HCV Infection

**Fig 7:**
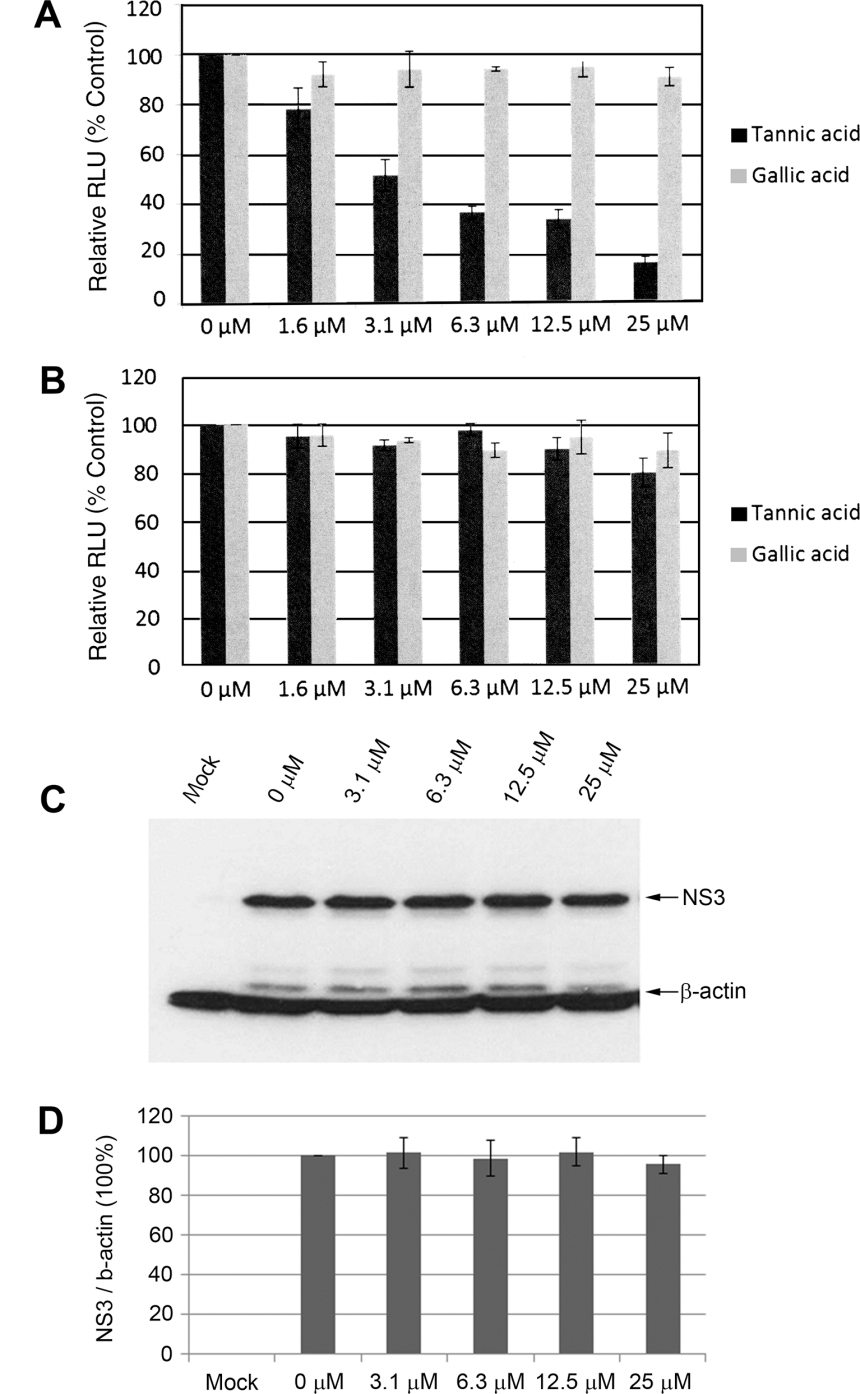
Comparison of the effect of gallic and tannic acid on HCV infection. (A) Effect during inoculation in an HCV cell culture system. Huh7.5 cells were infected with JFH1-AM120-Rluc for two hours in the presence of the specified concentrations of gallic or tannic acid, cells were washed and further incubated for 48 hours as described in Materials and Methods. Cells were lysed and luciferase activity was used to measure HCV infection as in Fig 2. Results are presented as the mean ± SD of relative light units (RLU) from three experiments. (B) Effect after inoculation in an HCV cell culture system. Huh7.5 cells were infected with JFH1-AM120-Rluc for two hours, cells were washed, gallic or tannic acid was added in the specified concentrations and cells were incubated for 48 hours. Cells were lysed and luciferase activity was measured as described previously. Results are presented as the mean ± SD of relative light units (RLU) from three experiments. (C) Effect in an HCV replicon system. HCV1b replicon RNA was electroporated into Huh7.5 cells, seeded into six-well plates and cultured for 48 hours. Cells were trypsinized, seeded in twelve-well plates, cultured overnight and gallic acid at concentrations of 0 μM, 3.1 μM, 6.3 μM, 12.5 μM and 25 μM was added at following day. The cells were cultured with gallic acid for 48 hours prior to analysis. Mock electroporation (no HCV1b replicon RNA) of cells served as controls. Cells were lysed and Western blots done with an anti-NS3 monoclonal antibody as described in Materials and Methods. Experiments were performed three times and representative examples are shown. (D) The levels of HCV NS3 protein were normalized to β-actin and the ratios of NS3/β-actin relative to a 0 μM tannic acid control (designated 100%) were plotted against inhibitor concentrations. The data are presented as mean ± SD (n = 3) of the NS3 levels normalized to β-actin for three independent experiments.

Tannic acid is relatively large molecule that can be broken down into smaller gallic acid and glucose components [25, 41]. To determine whether gallic acid also inhibited HCV entry in our system, Huh7.5 cells were infected with the HCV JFH1-AM120-Rluc in the presence of increasing concentrations of gallic acid. Tannic acid was also tested as a control. After two hours infection (37°C) the inoculum was removed, cells were washed and fresh complete medium was added. At 48 hours of incubation cells were lysed and luciferase activity was measured to assay for HCV infection. A dose-dependent decrease of Rluc activity was observed in tannic acid treated cells, but no effect was observed in gallic acid treated cells indicating that it did not inhibit HCV entry (Fig 7A). To determine if gallic acid inhibited HCV replication, Huh7.5 cells were infected with the HCV JFH1-AM120-Rluc for two hours, cells were washed and increasing concentrations of gallic acid (0 to 25 μM) were added. Tannic acid was used as a control. The cells were incubated for 48 hours and Rluc activity was measured. These results provide evidence that gallic acid had no detectable anti-HCV activity during HCV entry and replication steps at concentrations of 0 to 25 μM (Fig 7B).

Higher concentrations of gallic acid were tested in the HCV JFH1-AM120-Rluc cell culture system, during and after inoculation as above, and no inhibition was seen 50 μM (data not shown). Concentrations of gallic acid ≥100 μM produced confounding effects on the pH of the cell culture medium, making it difficult to determine a true IC_50_ and CC_50_. The effect of gallic acid was also tested using a sub-genomic HCV 1b replicon cell culture system following the methods described for tannic acid. As expected, gallic acid did not inhibit HCV replication in this system up to concentrations of 25 μM (Fig 7C and 7D).

## Discussion

Tannins are a unique group of phenolic *compounds* found in plants with molecular weights between 500 and 30,000 Da and are widely distributed in foods and beverages [25, 26]. Tannic acid, a member of this class of compounds, is present in many cereals, legumes, fruits, herbs and beverages such as tea, red wine, coffee [26–28]. Tannins in plants are believed to play an important role in protecting them against insect and microbial infections. Tannic acid exhibits a number of biological properties that have suggested health benefits and have been used to enhance farm animal grow [31, 38, 40]. To our knowledge, our results provide the first evidence that tannic acid is a potent inhibitor of HCV entry into Huh7.5 cells, possibly at the docking of the virus to the cell surface step, at relatively low concentrations (IC_50_ 5.8 μM). As expected from a viral cell entry inhibitor, tannic acid also blocked the cell-to-cell spread of HCV in culture. However, it did not inhibit HCV replication. Interestingly, tannic acid has been found to inhibit the attachment of other viruses to host cells, including norovirus binding to HBGA receptors and the attachment of influenza A virus to cells with an EC_50_ of 4.3 μM [39, 41]. The less widely distributed green-tea polyphenol tannin, epigallocatechin-3-gallate (EGCG), was demonstrated to inhibit HCV entry by blocking viral attachment to cells [42, 43]. The other tannins, such as EGCG, chebulagic acid and punicalagin have been suggested to have broad-spectrum antiviral activities [55]. Although the mechanisms of actions of polyphenols, such as tannic acid, have primarily been considered to be due to their antioxidant activity, we suggest that their ability to prevent the entry of viruses into cells is more likely due to their ability to form macromolecular complexes on the cell surface [56, 57]. It is interesting to speculate that naturally occurring tannins in food and beverages, when consumed in appropriate quantities, may sometimes provide a barrier to pathogenic microorganisms in both humans and other mammals.

Studies of the metabolism of tannic acid in rats, mice, sheep and chickens indicate that gallic acid, 4-O-methylgallic acid, pyrogallol, resorcinol, and ellagic acid are the major metabolites generated from tannic acid [58–62]. However, the concentrations tannic acid in blood after meals or oral dosing and its metabolism in humans remain relatively unstudied. Interestingly, tannic acid consumed at doses of 150 mg per day by type II diabetic patients significantly decreased blood glucose levels compared to controls [63]. Nevertheless, despite the interest in possible therapeutic applications of dietary tannic acid and other polyphenols their safe levels of consumption remain to be fully defined.

HCV entry is a complex multistep process involving multiple cell-surface proteins and post-binding events [12, 15, 64]. Viral entry is an essential role in the pathogenesis of HCV infection, especially in sustaining chronic infection and during the infection of donor livers transplanted into patients with chronic hepatitis C [65, 66]. HCV cell entry inhibitors, in combination with direct-acting antivirals, might provide an effective means of preventing the infection of transplanted livers in the many patients with chronic hepatitis C receiving transplants. The combined use of hepatitis B immune globulin, a well characterized HBV entry inhibitor, with nucleotide analogues has essentially eliminated HBV recurrence in liver transplantation patients [67]. Although a number of HCV cell entry inhibitors have been reported in different systems, they represent a relatively undeveloped area of HCV antivirals [21–24]. Our results provide a rationale for further exploring the cell entry HCV antiviral activity of tannic acid and similar polyphenols as possible therapeutics for chronic hepatitis C.
